# Identification of novel biomarkers and immune infiltration features of recurrent pregnancy loss by machine learning

**DOI:** 10.1038/s41598-023-38046-4

**Published:** 2023-07-03

**Authors:** Yujia Luo, Yuanyuan Zhou

**Affiliations:** 1 Department of NICU, Sir Run Run Shaw Hospital, School of Medicine, Zhejiang University, Hangzhou, China; 2 Department of Reproductive Endocrinology, Women’s Hospital, School of Medicine, Zhejiang University, Hangzhou, China

**Keywords:** Computational biology and bioinformatics, Immunology, Molecular biology

## Abstract

Recurrent pregnancy loss (RPL) is a complex reproductive disorder. The incompletely understood pathophysiology of RPL makes early detection and exact treatment difficult. The purpose of this work was to discover optimally characterized genes (OFGs) of RPL and to investigate immune cell infiltration in RPL. It will aid in better understanding the etiology of RPL and in the early detection of RPL. The RPL-related datasets were obtained from the Gene Expression Omnibus (GEO), namely GSE165004 and GSE26787. We performed functional enrichment analysis on the screened differentially expressed genes (DEGs). Three machine learning techniques are used to generate the OFGs. A CIBERSORT analysis was conducted to examine the immune infiltration in RPL patients compared with normal controls and to investigate the correlation between OFGs and immune cells. Between the RPL and control groups, 42 DEGs were discovered. These DEGs were found to be involved in cell signal transduction, cytokine receptor interactions, and immunological response, according to the functional enrichment analysis. By integrating OFGs from the LASSO, SVM-REF, and RF algorithms (AUC > 0.880), we screened for three down-regulated genes: ZNF90, TPT1P8, FGF2, and an up-regulated FAM166B. Immune infiltration study revealed that RPL samples had more monocytes (*P* < 0.001) and fewer T cells (*P* = 0.005) than controls, which may contribute to RPL pathogenesis. Additionally, all OFGs linked with various invading immune cells to varying degrees. In conclusion, ZNF90, TPT1P8, FGF2, and FAM166B are potential RPL biomarkers, offering new avenues for research into the molecular mechanisms of RPL immune modulation and early detection.

## Introduction

Recurrent pregnancy loss (RPL) is a distressing pregnancy disorder defined as the presence of two or more clinically recognized pregnancy losses before 20–24 weeks of gestation^1^. RPL affects about 2.5% of women who are trying to get pregnant. Recurrent miscarriages can be caused by a variety of conditions, including genetic, anatomical, endocrine, and immune-related illnesses^2^. However, the cause of about 50% of RPL instances is still unknown, and the etiology of RPL has not yet been thoroughly clarified^3^. As a result, progress in the development of accurate diagnosis and early prediction of recurrent miscarriage is stymied.

A woman's endometrial immune system is essential to the success of her pregnancy because it functions as a semi-allograft of the maternal host. Early in pregnancy, roughly 40% of the decidua’s cells are endometrium-resident immune cells, which serve regulatory roles during embryo implantation to guarantee maternal tolerance of the embryo^4^. Decidual lymphocytes play a crucial role in the early stages of pregnancy, among other things by removing apoptotic cells, spotting infections, encouraging trophoblast invasion, and controlling decidualization. Activated natural killer (NK) cells release growth-promoting factors that promote fetal maturation, and CD49a^+^ Emos^+^ NK cells recognize HLA-G expressed on extravillous trophoblasts^5,6^. Depletion of CD4+CD25+Treg cells results in pregnancy loss in mice because regulatory T (T reg) cells promote tolerance between fetal and maternal cells^4,7^. Furthermore, the quantity of tolerogenic dendritic cells (DCs) in the endometrium was dramatically decreased in the mid-luteal phase of RPL women, which induced the differentiation of Treg cells in the endometrium and other tissues^8,9^. At the same time, depletion of DCs in the endometrium also interferes with embryo implantation and leads to early embryo resorption, which is related to impaired decidualization and reduced vasodilation^10^. Circulating monocytes infiltrate into the decidua mediated by cytokines and chemokines. They differentiate into macrophages or DCs at the onset of pregnancy, participating in regulating maternal–fetal immunity^11^. Collectively, RPL is hypothesized to have a common etiology of compromised endometrial immunity.

Recently, major functional genes in several diseases have been discovered using microarray technology and thorough bioinformatics analysis, which can then be employed as diagnostic and predictive biomarkers^12–14^. Finding illness biomarkers is frequently done using machine learning (ML) techniques. We can account for the magnitude and direction of interactions between predictors and outcomes using Support Vector Machines-Recursive Feature Elimination (SVM-RFE) in machine learning^15^. A gene expression-based deconvolution technique called CIBERSORT is employed to evaluate immune cell infiltration^16^. To the best of our knowledge, however, the combination study of SVM-RFE, LASSO, Random Forest (RF), and CIBERSORT has not been used to identify putative biomarkers of RPL and forecast immune cell infiltration in RPL patients.

The purpose of this study was to screen for novel biomarkers in the endometrium associated with RPL using ML techniques. In addition, we used the CIBERSORT algorithm to assess immune cell infiltration in RPL and analyzed the relationship between biomarker expression and immune cell infiltration.

## Materials and methods

### Preprocessing and collection of data

**Figure 1:**
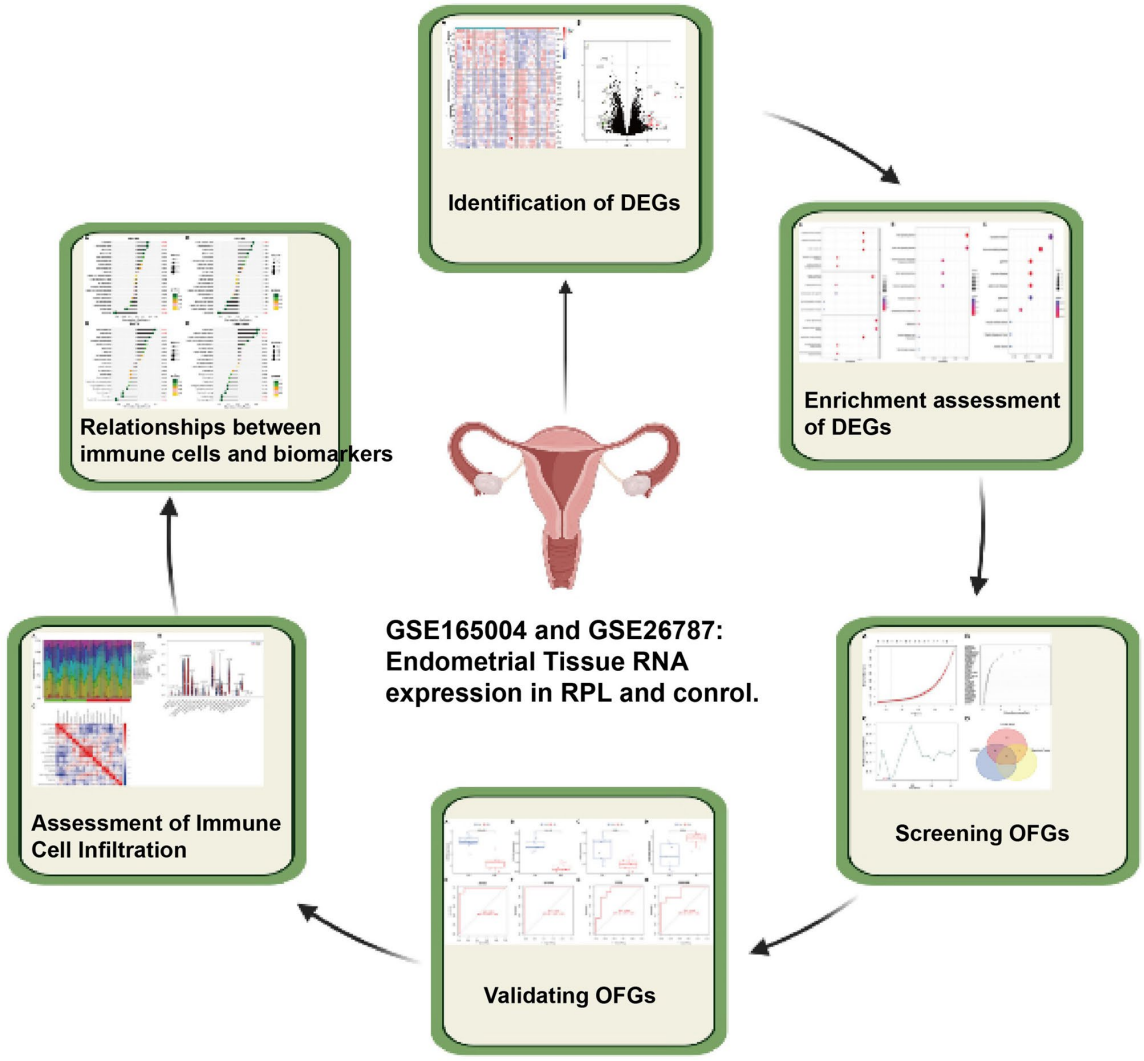
The workflow of the study.

In Fig. 1, we can see the workflow of the research. GSE165004 and GSE26787 were downloaded from the Gene Expression Omnibus (GEO) database in NCBI^17^. GSE165004 was based on the GPL16699 platform, which contained endometrial tissues of 24 RPL women and 24 controls. And GSE26787 was based on GPL570, consisting of 5 RPL patients and 5 control endometrial samples. With R packages “limma” and “sva”, two datasets were then merged and batch-normalized^18^. With the help of R software, 40 RPL and 18 healthy women were randomly divided into the training and testing cohorts in a ratio of two to one for the following analysis.

### Calculation of differentially expressed genes

Using the "limma" R package, we gained differences in gene expression between RPL and control tissues, and DEGs were set to |log2FC|> 1.0 along with *P*-value < 0.05. For visualizations of DEGs, heatmaps and volcano graphs were produced by "ggplot2" and "pheatmap" packages in R.

### Enrichment assessment of DEGs

The Gene Ontology (GO), Disease Ontology (DO) and Kyoto Encyclopedia of Genes and Genomes (KEGG) pathways^19^ were assessed for functional enrichment using the "clusterProfiler" package of R to discover the underlying biological functions of DEGs.

### Selection of optimal feature genes (OFGs)

To identify OFGs, we selected three different machine learning (ML) algorithms. "Glmnet", an R package, constructed the Least absolute shrinkage and selection operator (LASSO) binary logistic regression model. The optimal penalty parameter was determined and used in every signature using a tenfold cross-validation minimum^20^. The SVM-RFE, a nonlinear support vector machine implemented in the R package "e1071", "kernlab", and "caret", is applied to determine the OFGs^15^. To filter OFGs, we used the R package "randomforest" to generate 500 trees for every datapoint and retained the top 5 key genes^21^. Furthermore, the venn graph exhibited the OFGs of the intersection of three machine learning.

### Diagnostic quality verification of OFGs

A testing set was applied to verify screened OFGs as a validation step. To visualize the expression of crucial OFGs in the RPL and control women of testing set, we constructed boxplots using the R packages "ggplot2″ and "ggpubr”. Area under curve (AUC) was applied to assess the predictive value of OFGs using the receiver operating characteristic (ROC) curve computed by the "pROC" package in R^22^. Considering this, OFGs were recognized as prospective biomarkers with highly predictive and diagnostic capabilities once the AUC exceeded 0.85.

### Assessment of immune cell Infiltration

Utilizing the CIBERSORT algorithm (https://cibersort.stanford.edu/), we computed the infiltrating abundances and differences of 22 immune cells. The outcomes were visualized with heatmap, and violin graph produced with the "corrplot" and "ggplot2" packages in R^16^. The relationship between OFGs and immune cells was estimated by spearman correlation coefficient.

### Statistical analysis

Every statistical calculation and graph were executed by R (version 4.2.2). It was confirmed once p-value less than 0.05 as statistically significant.

## Results

### Identification of DEGs

**Figure 2:**
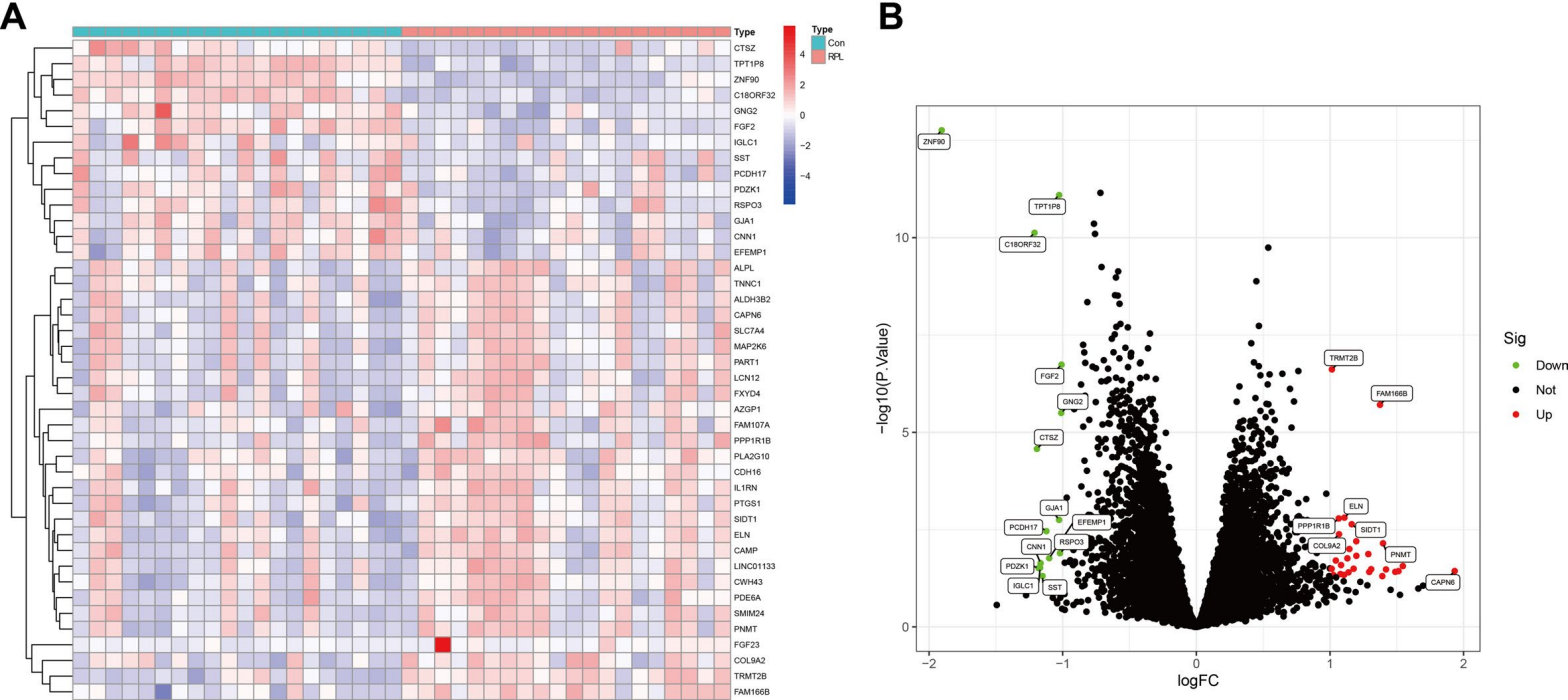
Differentially expressed genes (DEGs) identified between RPL and control women. (**A**) Heatmap. (**B**) Volcano plot.

Here is a diagram of the study's workflow (Fig. 1). DEGs were generated based on the training dataset, which contained endometrium tissues from 20 RPL patients and 20 controls. A comparison between RPL and control women revealed 42 DEGs with 28 upregulated and 14 downregulated genes. Heatmaps and volcano graphs were produced correspondingly to display the consequences (Fig. 2).

### Enrichment assessment of DEGs

**Figure 3:**
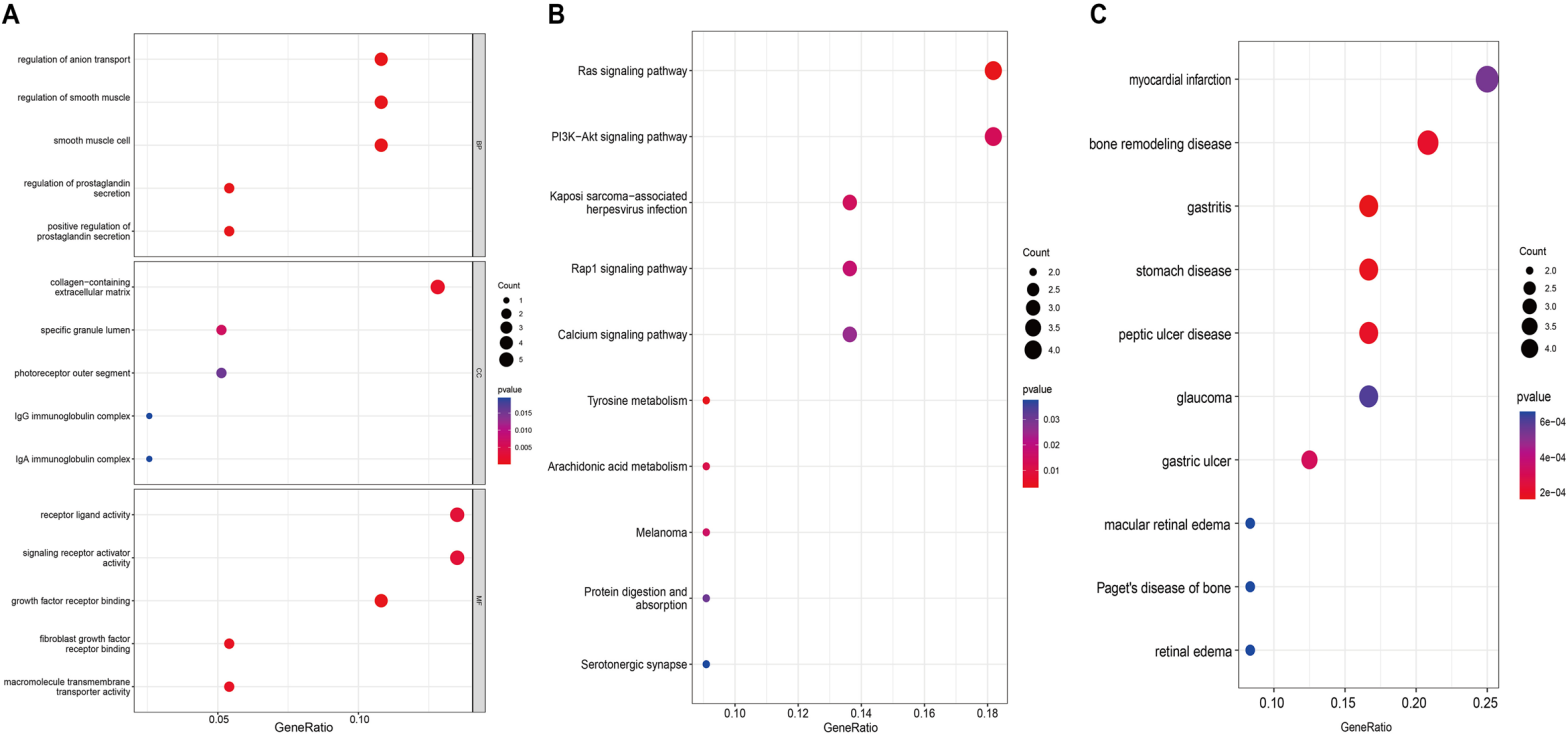
Functional enrichment analysis of DEGs. (**A**) GO analysis was executed to identify the potential functions of DEGs, containing CC, MF, and BP. (**B**) KEGG pathway was evaluated between RPL and control patients regarding DEGs. (**C**) DO analysis was used to evaluate the enrichment of DEG in the disease.

To complete the enrichment analysis of DEGs by GO/DO/KEGG, we applied the "clusterProfiler" package in R. For GO analysis, In RPL patients, the biological process, cellular component, and molecular function associated with anion transport, collagen–containing extracellular matrix and receptor-ligand activity were identified as the most enriched functions (Fig. 3A). KEGG analysis primarily targeted on Ras signalling pathway, and PI3K–Akt signalling pathway (Fig. 3B). Furthermore, DO analysis revealed tight associations of DEGs with myocardial infarction, bone remodelling disease and peptic ulcer disease in RPL women (Fig. 3C).

### Screening and validating OFGs

**Figure 4:**
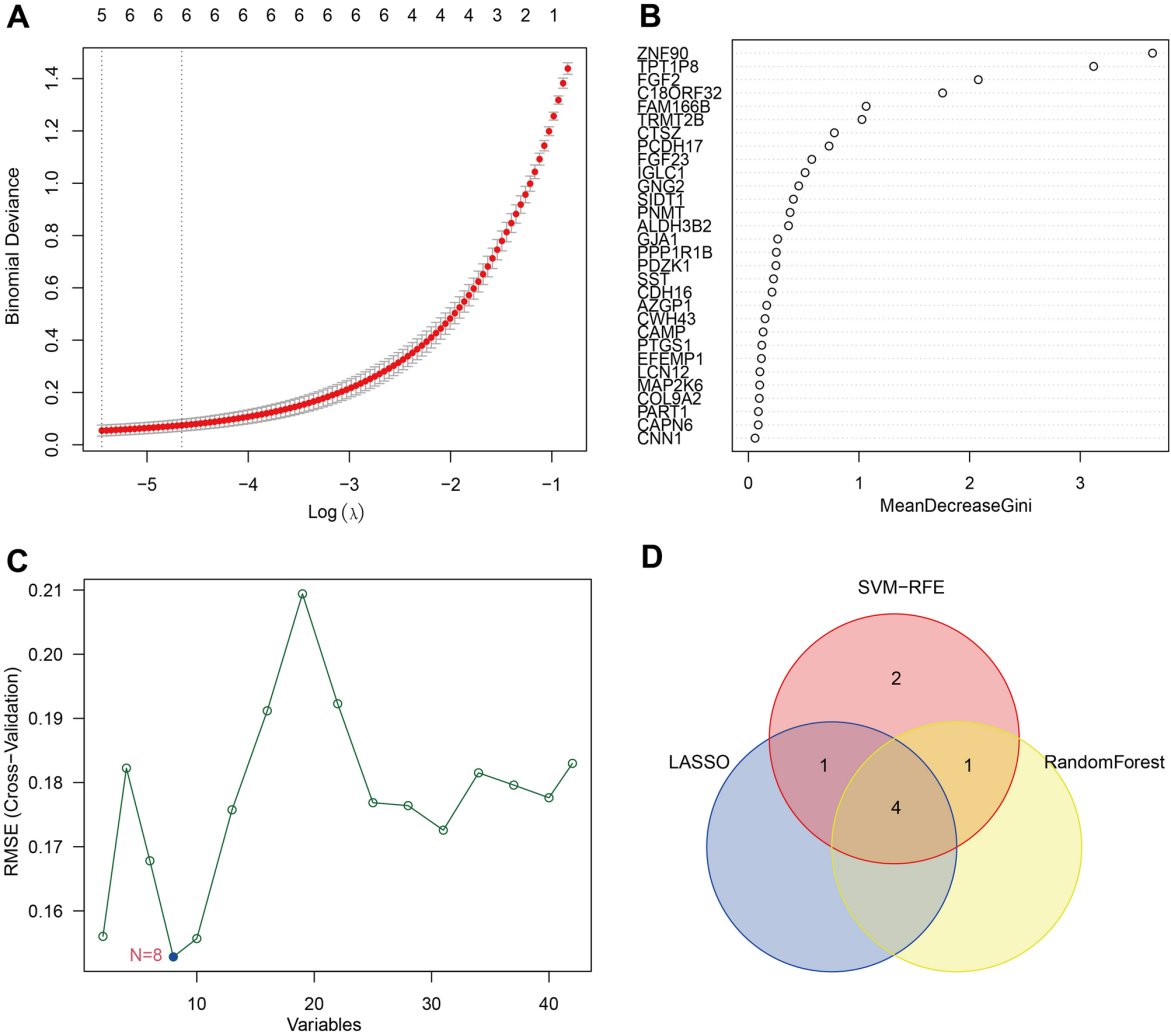
Screening underlying OFGs by machine learning. (**A**) Identifying biomarkers by LASSO algorithm. (**B**) Random Forest algorithm treated the top 5 genes in terms of MeanDecreaseGini score as OFGs. (**C**) SVM-RFE algorithm filters out 8 OFGs. (**D**) Venn diagram displaying four OFGs intersected by machine learning algorithms.

**Figure 5:**
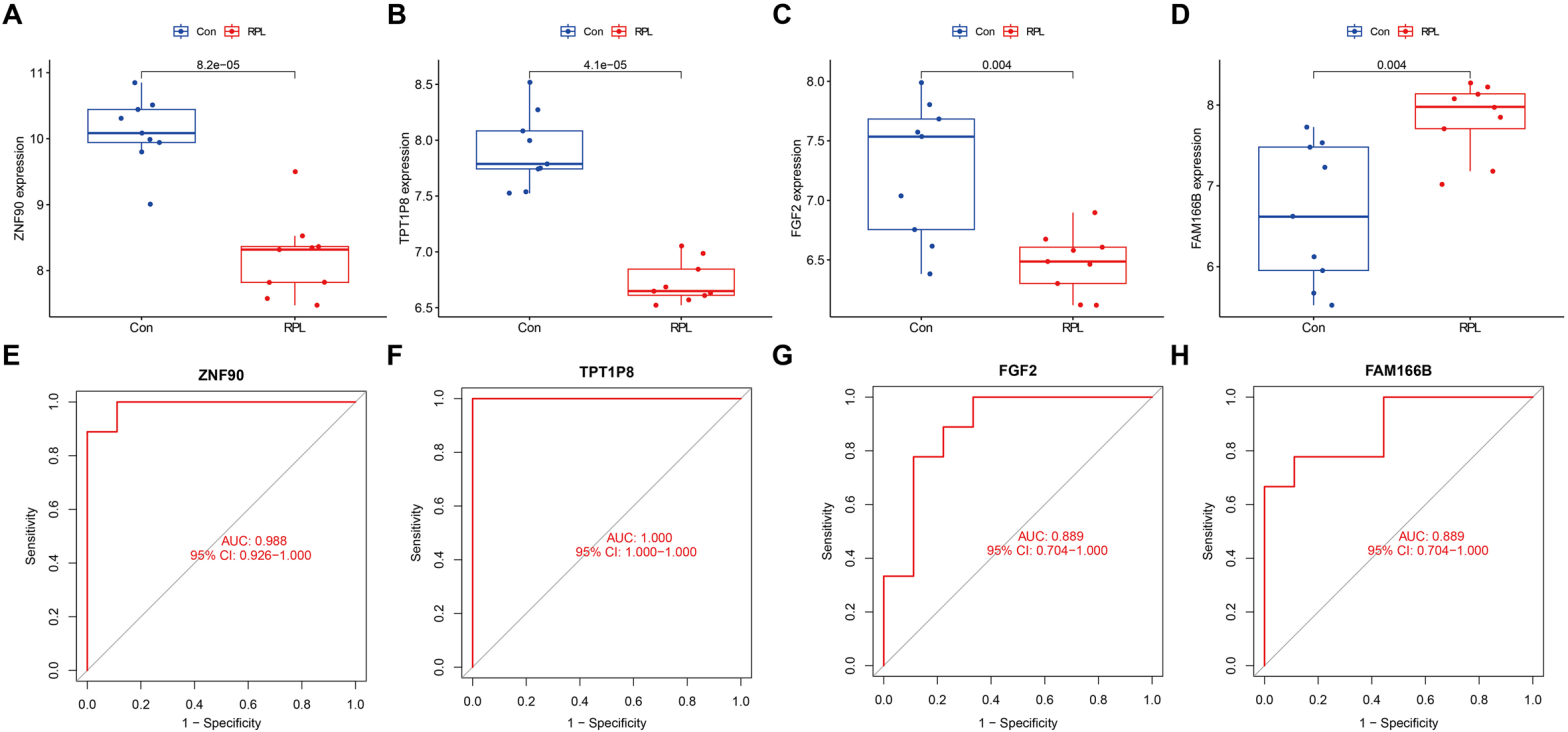
Validation of the OFGs. (**A**–**D**) Expression of ZNF90, TPT1P8, FGF2 and FAM166B in RPL patients compared to controls. (**E**–**H**) Diagnostic effectiveness of ZNF90, TPT1P8, FGF2 and FAM166B in ROC curves.

In the RPL-related DEGs, the LASSO and RF algorithms each screened five valuable genes. Additionally, the SVM-REF algorithm was applied to filter six crucial genes. The four intersecting OFGs are known as zinc finger protein 90 (ZNF90), putative translationally controlled tumor protein-like protein TPT1P8 (TPT1P8), fibroblast growth factor 2 (FGF2) and family with sequence similarity 166, member B (FAM166B) (Fig. 4). In the RPL, ZNF90, TPT1P8, and FGF2 are down-regulated and FAM166B is up-regulated. As verified by the testing set, the expression of OFGs were noticeably decreased in RPL women, except for FAM166B (Fig. 5A–D). To assess their diagnostic effectiveness, we produced ROC curves for the OFGs in testing cohort. All OFGs displayed excellent diagnostic results with AUCs exceeding 0.88 (Fig. 5E–H). Consequently, ZNF90, TPT1P8, FGF2 and FAM166B were identified to be promising biomarkers for diagnosing RPL.

### Assessment of immune cell infiltration

**Figure 6:**
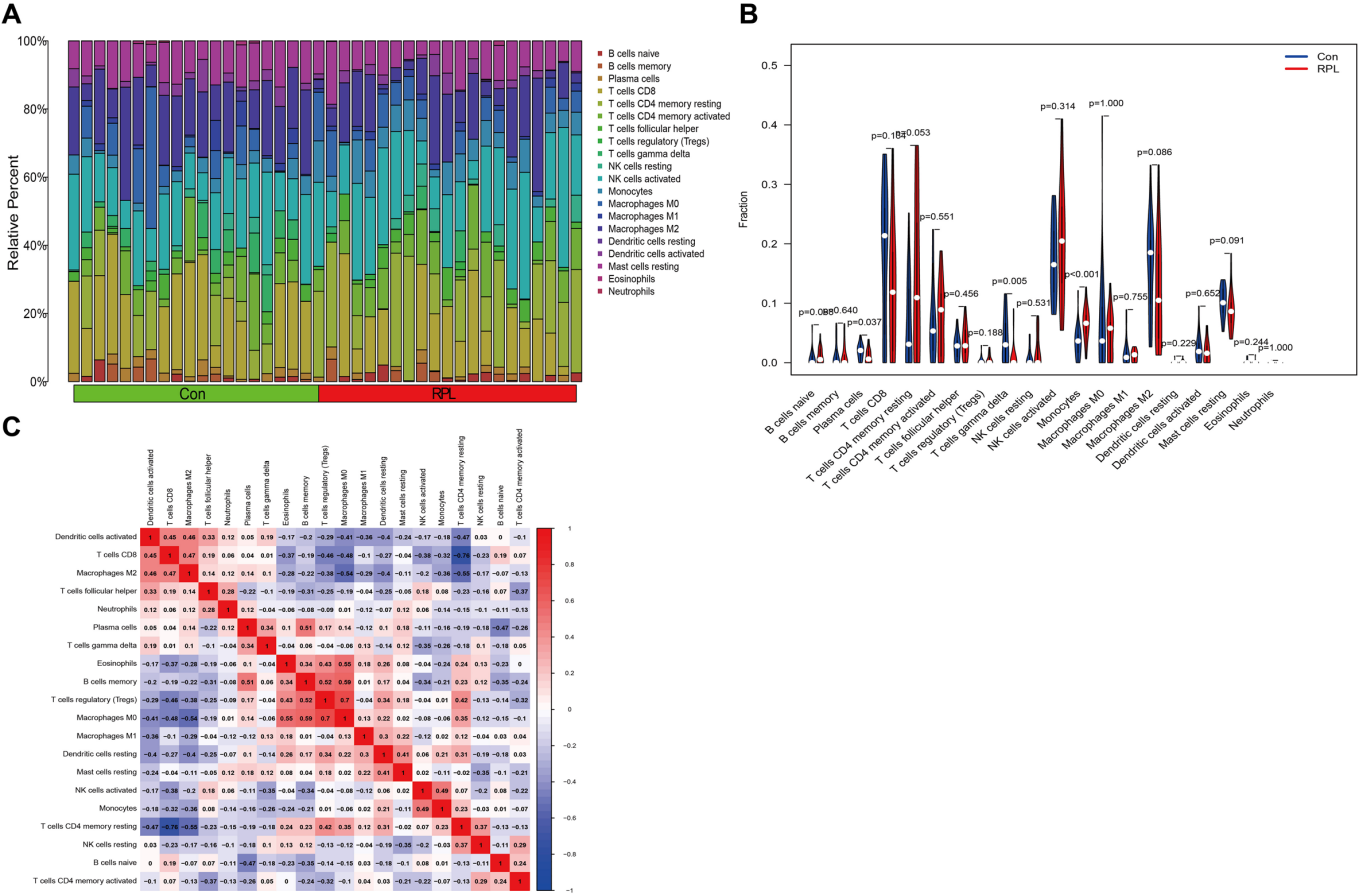
Proportion and association of immune cell infiltration. (**A**) The ratio of 22 immune cell subtypes between RPL and controls women. (**B**) Violin diagram showing differences in immune cells between RPL and controls women. (**C**) Correlation analysis among 22 immune cells.

In further analysis, we applied the CIBERSORT algorithm to discover the pertinent proportions of 22 different immune cell types. As shown in the bar chart, each sample has a different proportion of immune cell subpopulations (Fig. 6A). The violin graph demonstrated that infiltration of monocytes was strongly significant in the RPL group, whereas infiltration of γδ T cells exhibited remarkably significant in control dataset (Fig. 6B). Moreover, the interaction among immune cells revealed that regulatory T cells exhibited the most positive correlation with M0 macrophages, although CD8 T cells negatively exhibited relationship with CD4 memory resting T cells (Fig. 6C).

### Relationships between immune cells and biomarkers

**Figure 7:**
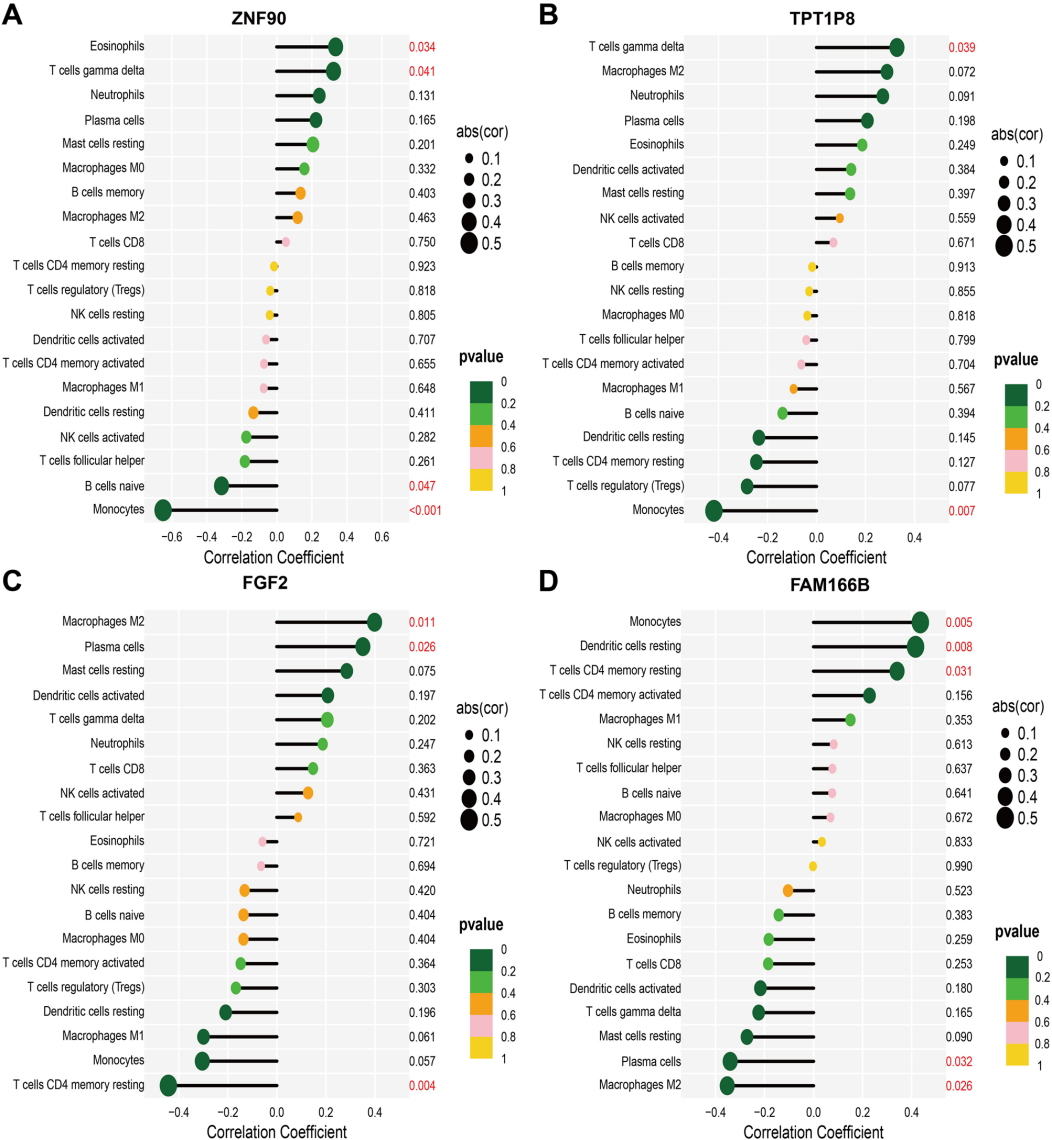
Visualization of Spearman correlation between 4 OFGs and immune cells in RPL patients. (**A**) ZNF90. (**B**) TPT1P8. (**C**) FGF2. (**D**) FAM166B.

Correlation analyses were executed to estimate the connections between immune cells and biomarkers. We discovered that the down-regulated genes: ZNF90 and TPT1P8 were positively related to γδ T cells (ZNF90: R = 0.324, *P* = 0.041; TPT1P8: R = 0.328, *P* = 0.034), but negatively associated with monocytes (ZNF90: R = -0.649, *P* < 0.001; TPT1P8: R =  − 0.418, *P* = 0.007). M2 macrophages and plasma cells were positively linked to down-regulated FGF2 and negatively connected to up-regulated FAM166B, whereas CD4 resting memory T cells were inversely related to these two genes. In addition, ZNF90 was also concerned with eosinophils and naive B cells; FAM166B was associated with monocytes and resting dendritic cells (Fig. 7).

## Discussion

RPL is still a major health concern in reproductive medicine, creating a significant psychological burden to individuals because 50% of RPL is idiopathic and evidence-based therapy is restricted. Currently, machine learning algorithms are excellent tools for analyzing underlying linkages and selecting ideal parameters for gene selection among all DEGs of biological significance in high-dimensional data. The discovery of new genes as potential biomarkers, as well as the study of immune cell infiltration features, will have a substantial impact on the early diagnosis and prediction of RPL. In this study, we identified a total of 42 DEGs, of which 28 genes were upregulated, and 14 were downregulated, based on the gene expression datasets of RPL and normal controls. Multifunctional enrichment analysis showed that these DEGs were related to MAPK signaling pathway, PI3K-Akt signaling pathway, inflammation and immune responses. Then based on three machine learning algorithms (LASSO regression model, RF algorithm and SVM-RFE algorithm), we screened out 4 best eigengenes (FGF2, FAM166B, ZNF90 and TPT1P8). Finally, we revealed the relationship between the 4 OFGs and immune cells using the CIBERSORT algorithm.

Fibroblast growth factor (FGF) regulates cell fate, angiogenesis, immunity, and metabolism through signalling through its receptors FGFR1, FGFR2, FGFR3 or FGFR4. According to research, dysregulation of FGF signaling causes human diseases such as lung, breast, and stomach cancer, as well as achondroplasia^23^. Furthermore, FGF and its receptors are major factors in fetal and placental angiogenesis. The FGF signaling process regulates immunity dynamically as well as regulating inflammation and tissue repair by immune cells. After FGFR1/2 signaling and VEGF/ANGPT2 secretion, FGF2 can promote endothelial cell proliferation and migration, for example^24,25^. FGF1 and FGF2 promote neutrophil chemotaxis to damaged tissues through FGFR2 signaling^26^. In addition, *Cox* reported that implantation of exogenous FGF-2 into the quail embryonic environment induced angiogenic cells and patterned blood vessel formation^27^. Thus, low FGF2 expression may contribute to RPL by hindering embryonic angiogenesis. FAM166B is a gene that has yet to be studied in depth. Previous studies involving FAM166B, which focused more on expression in multiple symmetric lipidosis and skeletal muscle, showed that FAM166B is highly expressed in adrenal glands and ciliated cells, but its precise function remains unclear^28^. A recent study showed that FAM166B expression correlates with breast cancer prognosis. The study found that the expression level of FAM166B in breast cancer was negatively correlated with the level of macrophage infiltration and positively correlated with the expression of CD 4+ T cells, which suggesting that the recruitment and regulation of immune infiltrating cells may be mediated by FAM166B in breast cancer^29^. Zinc finger proteins (ZFPs) are the largest family of transcription factors characterized by finger-like DNA-binding domains that play an important role in metabolic processes, autophagy, apoptosis, immune responses, differentiation, and stem cell maintenance^30^. However, only a few studies have reported the involvement of ZFPs in immune-related processes, such as immune response, immune homeostasis, and cytokine production recently^31,32^. ZFPs bind to Zinc, which is involved in the developmental process of oocytes. *Abu-Soud* reported that zinc deficiency leads to high ROS production in oocytes, which affects oocyte quality and female fertility by interfering with physiological antioxidant mechanisms that act on biomolecular, protein and cellular processes^33^. In the present study, low expression of ZFPs caused a decrease in binding efficiency to zinc, which may lead to RPL. Currently, there are few studies on TPT1P8, also known as FKSG2. The latest literature found that the significantly lower expression of TPT1P8 in the anterior cingulate cortex (ACC) of Cushing's disease (CD) patients was associated with immune function^34^. Accordingly, the OFGs screened in this study are involved in signaling transduction, inflammation and immune responses, which may contribute to RPL occurrence and progression.

Based on the CIBERSORT analysis, we found that RPL and the control group had significantly different levels of immune cell infiltration, especially monocytes and γδ T cells. Our study found that RPL samples had higher levels of monocyte infiltration. Consistent with our conclusions, previous studies also showed that women with RPL had higher monocyte concentrations detected in peripheral blood than normal fertile controls^35^. During normal pregnancy, immune cells at the fetal-maternal interface increase, such as uterine NK cells and macrophages. Monocytes are short-lived cells that arise from monocyte precursors in the bone marrow and makeup approximately 5–10% of the total number of circulating white blood cells^36^. Accumulating evidence suggests that circulating monocytes are recruited to the decidua at the onset of pregnancy to generate macrophages with essential immune functions. Thus, decidual macrophages contribute to maternal tolerance to fetal antigens^11^. Monocytes also present antigens to T cells, which regulate the adaptive immune response. In addition, they are involved in fundamental processes of a successful pregnancy, such as trophoblast invasion and tissue and vascular remodeling^37^. In addition, our results also showed that γδ T cells were decreased in RPL samples compared with normal controls. According to the T cell receptor (TCR), T cells are divided into αβ T and γδ T cells, which express αβ TCR and γδ TCR, respectively^38^. γδ T cells play numerous roles in establishing and maintaining immune tolerance in early pregnancy but are often overlooked. γδT cells are increased in the early decidua of normal pregnancy. They secrete anti-inflammatory cytokines such as IL-10 and TGF-β and transduce negative signals by expressing regulatory molecules such as PD-1, Tim-3, and CD 160^39,40^. From this, we speculate that dysregulation of endometrial monocytes and γδ T cells in women with RPL biases the maternal immune system towards pro-inflammatory properties, which may ultimately lead to RPL.

In our study, according to the correlation analysis, the four characteristic genes screened are related to immune cell infiltration of RPL. In Fig. 7, the expression of four candidate genes showed strong correlation with monocytes, but weak correlation with other differentially infiltrated immune cells. Comins-Boo suggests that monocyte dysregulation is a major factor contributing to RPL. We therefore hypothesize that key genes interacting with monocytes may promote the development of RPL. However, the specific mechanism for the weak correlation of γδ T cells and plasma cells with key genes is unknown and may be related to the small sample size.

The integration of microarray technology, bioinformatics analysis, and ML algorithms has become a hotbed for biomarker screening, diagnosis prediction, and prognosis evaluation of complicated diseases in recent years. Moreover, computational biology methods can provide the basis for further basic experimental design. In this study, the combination of the LASSO model, RF algorithm and SVM-RFE algorithm was applied to identify potential biomarkers of RPL, as it has rarely been done before. This study, however, has limited data, and more external data, clinical samples, and prospective clinical trials are needed in the future to verify the results.

## Conclusion

In this study, we found that ZNF90, TPT1P8, FGF2 and FAM166B could serve as candidate biomarkers for RPL, and we explored their correlations with immune cells in the pathogenesis of RPL. In addition, the differential infiltration of monocytes and γδ T cells is related to the onset and progression of RPL. Future studies with larger sample sizes and more predictive clinical measures are necessary for validating these results.

## Data Availability

The datasets supporting the conclusions of this article are available in the GEO database, including GSE165004 (https://www.ncbi.nlm.nih.gov/geo/query/acc.cgi?acc=GSE165004) and GSE26787 (https://www.ncbi.nlm.nih.gov/geo/query/acc.cgi?acc = GSE26787).

## References

[1] Bender Atik R, Christiansen OB, Elson J, Kolte AM, Lewis S, RPL EGGo (2018). ESHRE guideline: Recurrent pregnancy loss. Hum. Reprod. Open..

[2] Dimitriadis E, Menkhorst E, Saito S, Kutteh WH, Brosens JJ (2020). Recurrent pregnancy loss. Nat. Rev. Dis. Prim..

[3] van Dijk MM, Kolte AM, Limpens J, Kirk E, Quenby S, van Wely M (2020). Recurrent pregnancy loss: Diagnostic workup after two or three pregnancy losses? A systematic review of the literature and meta-analysis. Hum Reprod Update..

[4] Ticconi C, Pietropolli A, Di Simone N, Piccione E, Fazleabas A (2019). Endometrial immune dysfunction in recurrent pregnancy loss. Int. J. Mol. Sci..

[5] Fu B, Zhou Y, Ni X, Tong X, Xu X, Dong Z (2017). Natural killer cells promote fetal development through the secretion of growth-promoting factors. Immunity.

[6] Gamliel M, Goldman-Wohl D, Isaacson B, Gur C, Stein N, Yamin R (2018). Trained memory of human uterine NK cells enhances their function in subsequent pregnancies. Immunity.

[7] Aluvihare VR, Kallikourdis M, Betz AG (2004). Regulatory T cells mediate maternal tolerance to the fetus. Nat. Immunol..

[8] Lu Y, Giver CR, Sharma A, Li JM, Darlak KA, Owens LM (2012). IFN-gamma and indoleamine 2,3-dioxygenase signaling between donor dendritic cells and T cells regulates graft versus host and graft versus leukemia activity. Blood.

[9] Li Y, Wang R, Wang M, Huang W, Liu C, Fang Z (2021). RNA sequencing of decidua reveals differentially expressed genes in recurrent pregnancy loss. Reprod. Sci..

[10] Plaks V, Birnberg T, Berkutzki T, Sela S, BenYashar A, Kalchenko V (2008). Uterine DCs are crucial for decidua formation during embryo implantation in mice. J. Clin. Invest..

[11] Nagamatsu T, Schust DJ (2010). The immunomodulatory roles of macrophages at the maternal-fetal interface. Reprod. Sci..

[12] Na Z, Guo W, Song J, Feng D, Fang Y, Li D (2022). Identification of novel candidate biomarkers and immune infiltration in polycystic ovary syndrome. J. Ovarian Res..

[13] Tian Y, Lu Y, Cao Y, Dang C, Wang N, Tian K (2022). Identification of diagnostic signatures associated with immune infiltration in Alzheimer's disease by integrating bioinformatic analysis and machine-learning strategies. Front. Aging Neurosci..

[14] Huang KK, Zheng HL, Li S, Zeng ZY (2022). Identification of hub genes and their correlation with immune infiltration in coronary artery disease through bioinformatics and machine learning methods. J. Thorac. Dis..

[15] Mi X, Zou B, Zou F, Hu J (2021). Permutation-based identification of important biomarkers for complex diseases via machine learning models. Nat. Commun..

[16] Newman AM, Liu CL, Green MR, Gentles AJ, Feng W, Xu Y (2015). Robust enumeration of cell subsets from tissue expression profiles. Nat. Methods..

[17] Barrett T, Wilhite SE, Ledoux P, Evangelista C, Kim IF, Tomashevsky M (2013). NCBI GEO: Archive for functional genomics data sets–update. Nucl. Acids Res..

[18] Leek JT, Johnson WE, Parker HS, Jaffe AE, Storey JD (2012). The sva package for removing batch effects and other unwanted variation in high-throughput experiments. Bioinformatics.

[19] Kanehisa M, Goto S (2000). KEGG: kyoto encyclopedia of genes and genomes. Nucl. Acids Res..

[20] Frost HR, Amos CI (2017). Gene set selection via LASSO penalized regression (SLPR). Nucl. Acids Res..

[21] Kursa MB (2014). Robustness of Random Forest-based gene selection methods. BMC Bioinf..

[22] Robin X, Turck N, Hainard A, Tiberti N, Lisacek F, Sanchez JC (2011). pROC: An open-source package for R and S+ to analyze and compare ROC curves. BMC Bioinf..

[23] Katoh M (2016). Therapeutics targeting FGF signaling network in human diseases. Trends Pharmacol. Sci..

[24] Katoh M (2016). FGFR inhibitors: Effects on cancer cells, tumor microenvironment and whole-body homeostasis (Review). Int. J. Mol. Med..

[25] House SL, Castro AM, Lupu TS, Weinheimer C, Smith C, Kovacs A (2016). Endothelial fibroblast growth factor receptor signaling is required for vascular remodeling following cardiac ischemia-reperfusion injury. Am. J. Physiol. Heart Circ. Physiol..

[26] Haddad LE, Khzam LB, Hajjar F, Merhi Y, Sirois MG (2011). Characterization of FGF receptor expression in human neutrophils and their contribution to chemotaxis. Am. J. Physiol. Cell Physiol..

[27] Cox CM, Poole TJ (2000). Angioblast differentiation is influenced by the local environment: FGF-2 induces angioblasts and patterns vessel formation in the quail embryo. Dev. Dyn..

[28] Shamseldin HE, Shaheen R, Ewida N, Bubshait DK, Alkuraya H, Almardawi E (2020). The morbid genome of ciliopathies: An update. Genet. Med..

[29] Zhou, Y., Zhu, H., He, G., Zhang, H., Cheng, X., Liu, X. Overexpressed FAM166B predicts favorable prognosis and associated with metabolic pathways and tumor immune infiltrates in BRCA. (2021).

[30] Li X, Han M, Zhang H, Liu F, Pan Y, Zhu J (2022). Structures and biological functions of zinc finger proteins and their roles in hepatocellular carcinoma. Biomark Res..

[31] Carpenter S, Ricci EP, Mercier BC, Moore MJ, Fitzgerald KA (2014). Post-transcriptional regulation of gene expression in innate immunity. Nat. Rev. Immunol..

[32] Medzhitov R, Horng T (2009). Transcriptional control of the inflammatory response. Nat. Rev. Immunol..

[33] Camp, O. G., Bembenek, J. N., Goud, P. T., Awonuga, A. O., Abu-Soud, H. M. The implications of insufficient zinc on the generation of oxidative stress leading to decreased oocyte quality. *Reprod. Sci.* (2023). 10.1007/s43032-023-01212-0 PMC11047769 36920672

[34] Bauduin S, den Rooijen ILB, Meijer M, van der Werff SJA, Keo A, Dzyubachyk O (2021). Potential associations between immune signaling genes, deactivated microglia, and oligodendrocytes and cortical gray matter loss in patients with long-term remitted Cushing's disease. Psychoneuroendocrinology.

[35] Comins-Boo A, Valdeolivas L, Perez-Pla F, Cristobal I, Subhi-Issa N, Dominguez-Soto A (2022). Immunophenotyping of peripheral blood monocytes could help identify a baseline pro-inflammatory profile in women with recurrent reproductive failure. J. Reprod. Immunol..

[36] Faas MM, de Vos P (2017). Uterine NK cells and macrophages in pregnancy. Placenta.

[37] Abu-Raya B, Michalski C, Sadarangani M, Lavoie PM (2020). Maternal immunological adaptation during normal pregnancy. Front. Immunol..

[38] Xu QH, Liu H, Wang LL, Zhu Q, Zhang YJ, Muyayalo KP (2021). Roles of gammadeltaT cells in pregnancy and pregnancy-related complications. Am. J. Reprod. Immunol..

[39] Talukdar A, Rai R, Aparna Sharma K, Rao DN, Sharma A (2018). Peripheral Gamma Delta T cells secrete inflammatory cytokines in women with idiopathic recurrent pregnancy loss. Cytokine.

[40] Polgar B, Barakonyi A, Xynos I, Szekeres-Bartho J (1999). The role of gamma/delta T cell receptor positive cells in pregnancy. Am. J. Reprod. Immunol..

